# The Desire for Being Liked and the Fear of Negative Evaluation in Rhinoplasty Patients

**DOI:** 10.1007/s00266-024-03979-2

**Published:** 2024-03-26

**Authors:** Eda Albayrak, Nurcan Uzdil, Kerem Kökoğlu

**Affiliations:** 1 Department of Mental Health and Diseases Nursing, Faculty of Health Sciences, Erciyes University, Kayseri, Turkey; 2 Ear, Nose and Throat Diseases Department, Faculty of Medicine, Erciyes University, Kayseri, Turkey

**Keywords:** Desire for being liked, Fear of negative evaluation, Rhinoplasty

## Abstract

**Objective:**

The study was conducted to determine patients’ desire for being liked and the fear of negative evaluation before and after surgery.

**Materials and Method:**

The study was conducted quasi-experimentally using a one-group pretest-posttest (sixth month) design. The study population consisted of rhinoplasty patients hospitalized in the ear, nose, and throat service. The study was completed with 60 people. Participant information form, the desire for being liked scale, and the brief fear of negative evaluation scale were used as data collection tools. The data of the study were collected face-to-face between November 15, 2021, and March 15, 2023.

**Findings:**

It was determined that the mean scores of the desire for being liked were 19.43 ± 7.44 in the pretest and 12.15 ± 4.76 in the posttest, and the difference between the measurements was highly significant. Furthermore, it was determined that the mean score of fear of negative evaluation was 27.78 ± 9.81 in the pretest and 17.72 ± 6.91 in the posttest, and the difference between the measurements was highly significant. It was observed that there was a high, statistically significant, and positive relationship between the pretest and posttest fear of negative evaluation and desire for being liked scores.

**Conclusion:**

Patients’ desire for being liked and fear of negative evaluation, which were high before surgery, decreased significantly six months after surgery.

**Level of Evidence IV:**

This journal requires that authors assign a level of evidence to each article. For a full description of these evidence-based medicine ratings, please refer to the Table of contents or the online instructions to authors www.springer.com/00266.

## Introduction

Rhinoplasty, which is one of the most preferred aesthetic surgical interventions, is one of the most frequently performed aesthetic operations to eliminate deformities and dysfunctions of the nose [1]. It has been reported that only 10% of rhinoplasties performed worldwide are related to function, while the rest aim for visual change [2]. On the other hand, the high rate of psychiatric diagnoses among rhinoplasty patients draws attention. For instance, in a study conducted in Iran, 41% of the rhinoplasty patient group met psychopathological diagnoses such as body dysmorphic disorder (BDD), obsessive-compulsive disorder (OCD), social anxiety, generalized anxiety disorder, and somatic delusions [1, 3]. It is seen that most people who have aesthetic surgery have a self-image related to physical appearance and therefore seek surgical change [4]. Therefore, it can be said that human beings, who are social beings, care about the impression they leave on others. According to Yousefi et al. [5], individuals make intense efforts to make an effective impression on others and to look attractive. This effort can be manifested in expensive clothes, make-up, aesthetic operations, and attempts to intervene in the body. Çepikkurt and Coşkun [6] emphasized that the effort of individuals to look more beautiful or attractive stems from the concern about being disliked and some people are more concerned about this issue than others. The desire for being liked is a motive arising from the need for love, respect, acceptance, and feeling successful and sufficient [7]. The desire for being liked may bring with it the fear of being evaluated negatively. It is seen that individuals with a high desire for being liked are more sensitive to the evaluations of others and make more effort to be liked [8]. It was also stated that individuals with high social anxiety have a strong core belief that they will be evaluated negatively in social environments [9]. It was reported that individuals who care about the impression they leave on the people they communicate with and whether they are approved by these people exhibit various behaviors/initiatives to avoid creating a negative impression [10]. Rhinoplasty, one of these interventions, is the most commonly performed plastic surgery in Turkey [11]. Applications are made to this surgery for nasal breathing or aesthetic purposes. Before applying for surgery, the positive and negative consequences of the procedure should be investigated in detail and individuals should be questioned psychologically [11].

It has been determined that there are few psychosocial studies in this patient group in the literature, and it seems that studies on social appearance anxiety have been addressed more [12, 13]. For instance, Yigman and Inan [13] collected data after three months and reported that psychological factors such as appearance anxiety may act as a bridge between patient satisfaction and rhinoplasty results. Moreover, in the study conducted by Tulacı and Arslan [12], it was determined that individuals with high social appearance anxiety in the preoperative evaluation had less satisfaction in the 6th month after surgery. It is thought that these studies are mostly related to social appearance anxiety, and different psychosocial data will support the literature. There are no studies in the literature that examine the desire to be liked and the fear of negative evaluation together in rhinoplasty patients. It is thought that this study will be the first study to address the desire to be liked and the fear of negative evaluation in this group of patients with a long follow-up (six months) through a pre-test, post-test quasi-experimental model and will add originality to sthe literature. Therefore, this study was conducted to determine patients’ desire for being liked and fear of negative evaluation before and after surgery.

### Hypotheses of the Study

#### H1

There is a significant difference in desire for being liked scores before and after surgery in rhinoplasty patients.

#### H2

There is a significant difference in fear of negative evaluation scores before and after surgery in rhinoplasty patients.

## Materials and Methods

### Research Design

This study was conducted quasi-experimentally in a one-group pretest-posttest design to determine the desire for being liked and fear of negative evaluation before and after surgery in patients undergoing rhinoplasty surgery.

### Study Population and Sample

The population of the study consisted of rhinoplasty patients hospitalized in the ear, nose, and throat service. To determine the sample of the study, studies on the desire for being liked and fear of negative evaluation in rhinoplasty patients could not be reached. Studies that would support the hypotheses of the desire for being liked and fear of negative evaluation in our study were also not found. A pilot study was needed for this. Sim and Lewis applied the upper confidence limit approach in their study and determined that a pilot trial of 55 participants with a 95% upper confidence limit would minimize the overall sample size for small to medium standardized effect sizes (0.2–0.6). Sim and Lewis’ 95% upper confidence limit was preferred because it has an effect of increasing the required sample size estimates compared to Kieser and Wassmer for both pilot and main trials [14, 15]. Sim and Lewis [14] stated the minimum pilot sample size as 55 and above. Accordingly, the minimum sample size for our study was planned to be 55 people, and considering the possible loss, a total of 65 people were invited by taking 20% more samples. Five of these patients were excluded from the study due to incomplete filling of the scale questions.

### Participants

The study included individuals who were 18 years of age or older, who would undergo rhinoplasty surgery, who did not have cognitive impairment, who could speak Turkish, and who volunteered to participate in the study. The data were collected face-to-face between November 15, 2021, and March 15, 2023.

### Data Collection Tools

#### Introductory Information Form

The diagnostic information form includes information such as age, gender, and chronic diseases of the patients.

#### The Desire for Being Liked Scale

The scale developed by Kaşıkara and Doğan [7] consists of nine items. There are no reverse items in the scale and the scale has a single-factor structure. The scale is graded on a four-point Likert scale (1 = Strongly Disagree, 4 = Strongly Agree). The lowest score that can be obtained from the scale is nine and the highest score is 36. The higher the scores obtained from the scale, the higher the desire to be liked. Cronbach’s alpha internal consistency coefficient of the scale is 0.81, while it was determined to be 0.97 in the present study.

#### The Brief Fear of Negative Evaluation Scale

The scale created by Leary [16] was adapted into Turkish by Çetin et al. [17]. In the scale, items 2, 6, and 9 are reverse coded and the scale consists of 11 items in total. The scale is graded on a five-point Likert scale (1 = Not at all appropriate, 5 = completely appropriate). The highest score that can be obtained from the scale is 55, and high scores indicate that the fear of negative evaluation is high. Cronbach’s alpha internal consistency coefficient of the scale is 0.84, while it was 0.96 in the present study.

### Data Collection

The data were collected by the researchers in two stages (pre-test and 6th month) by face-to-face interview technique. The purpose of the study was explained to the patients admitted to the hospital, questionnaires were given to the participants who agreed to participate, and the individuals filled out the forms themselves. For the pretest data, the introductory information form, the desire for being liked scale, and the brief fear of negative evaluation scale were used. Moreover, the desire for being liked and the brief fear of negative evaluation scales were administered to the patients at the sixth month measurements. During the answering period, the researchers were present with the individuals and answered the questions asked about the forms. It took approximately ten minutes to complete the forms.

### Data Evaluation

Data were evaluated in the IBM SPSS Statistics 21 statistical package program. Summary statistics of the variables were given as number of units (n), percentage (%), mean (*x̄*), and standard deviation (SD). In examining the relationships between variables, the Kolmogorov-Smirnov test was used to examine whether the variables met the normality assumption. According to the kurtosis and skewness coefficients of the variables and the coefficient of variation, the variables were determined to be normally distributed [18]. A paired two-sample *t*-test was applied to evaluate the dependent data in the pre-test and post-test measurements. Independent samples *t*-test was used for independent two-group measurements and ANOVA test was used for three-group measurements. Pearson’s test was used for correlation between variables. In comparisons, *p* < 0.05 was considered statistically significant.

### Ethical Dimension of the Research

Approval from the Ethics Committee of the relevant university (2021/731) and institutional permission from the relevant service were obtained for the research. Individuals were informed about the purpose of the study that participation in the study was voluntary, they could leave the study at any time, their names would be kept confidential, and consent was obtained.

## Findings

Table 1 shows that the mean age of the patients who participated in our study was 32.57 ± 11.61, 53.3% were female, 55% were married, 53.3% were higher education graduates, 56.7% had children, 33.3% were government employees, 71.7% had moderate income, 60% were not working, 78.3% lived in the provincial center, 60% had not undergone any previous surgery, 16.7% had a chronic disease, and 61.9% had undergone surgery for a deviated septum.

**Table 1:** Distribution of rhinoplasty patients according to descriptive characteristics

Characteristics	*n*	%
Age (Mean ± SD)	32.57 ± 11.61	
*Gender*		
Female	32	53.3
Male	28	46.7
*Education status*		
Primary education	11	18.3
Secondary education	17	28.3
Higher education	32	53.4
*Marital status*		
Married	33	55.0
Single	27	45.0
*Having child*		
Yes	34	56.7
No	26	43.3
*Working status*		
Working	24	40.0
Not working	36	60.0
*Income status*		
Low	15	25.0
Middle	43	71.7
High	2	3.3
*Place of residence*		
Province	47	78.3
District	10	16.7
Town	3	5.0
*Previous surgery status*		
Yes	24	40.0
No	36	60.0
*Profession*		
Government employee	20	33.3
Private sector	14	23.3
Housewife	15	25.1
Student	11	18.3
Reason for rhinoplasty surgery*		
Deviated septum	52	61.9
Not liking myself	25	29.8
Disliked by environment	7	8.3
*Presence of chronic disease*		
Yes	10	16.7
No	50	83.3

*Multiple responses

Table 2 shows the comparison of preoperative and postoperative scale mean scores in rhinoplasty patients. It was determined that the mean DBLS scores of rhinoplasty patients were 19.43 ± 7.44 in the pre-test and 12.15 ± 4.76 in the post-test and the difference between the measurements was highly significant (*p* < 0.001). Furthermore, the mean BFNE scores of rhinoplasty patients were 27.78 ± 9.81 in the pretest and 17.72 ± 6.91 in the posttest and the difference between the measurements was highly significant (*p* < 0.001).

**Table 2:** Comparison of preoperative and postoperative mean scale scores in rhinoplasty patients

Scales	Pre-test	Post-test	Difference	t*	*P*
	$\overline{x} \pm {\text{ss}}$	$\overline{x} \pm {\text{ss}}$			
DBLS	19.43 ± 7.44	12.15 ± 4.76	7.28 ± 8.18	6.900	**< 0.001**
BFNE	27.78 ± 9.81	17.72 ± 6.91	10.07 ± 11.38	6.854	**< 0.001**

Bold values indicate significant

*DBLS* desire for being liked scale, *BFNE* the brief fear of negative evaluation scale

*Paired-sample *t*-test

Table 3 shows the comparison of the preoperative and postoperative mean scale scores of the patients according to their descriptive characteristics. Statistically significant differences were obtained between the place of residence, presence of chronic disease, and pretest DBLS and BFNE mean scores (*p* < 0.05). It was determined that these significant differences were caused by those living in the city center and those without chronic disease, respectively. The posttest DBLS mean score was higher in those working in the private sector and this difference was significant (*p* < 0.05). Besides, the post-test BFNE mean score was higher in working people and this difference was significant (*p* < 0.05).

**Table 3:** Comparison of preoperative and postoperative mean scale scores in rhinoplasty patients according to descriptive characteristics

Features	*n*	Pre-test		Post-test	
		DBLS	BFNE	DBLS	BFNE
*Gender*					
Female	32	18.88 ± 8.22	26.64 ± 10.36	11.39 ± 3.81	16.91 ± 6.09
Male	28	20.11 ± 6.46	29.19 ± 9.09	13.07 ± 5.65	18.70 ± 7.81
Test stat.		0.350*	0.103*	− 0.992*	− 0.109*
*p*		0.728	0.918	0.327	0.914
*Education status*					
Primary education	11	16.73 ± 3.61	24.55 ± 4.34	10.82 ± 3.19	17.64 ± 6.83
Secondary education	17	19.41 ± 7.72	29.12 ± 10.64	12.88 ± 5.90	18.82 ± 8.15
Higher education	32	20.38 ± 8.18	28.19 ± 10.67	12.22 ± 4.57	17.16 ± 6.38
Test stat.		2.205**	1.953**	0.628**	0.316**
*p*		0.126	0.158	0.538	0.730
*Marital status*					
Married	33	19.75 ± 6.65	27.91 ± 9.55	11.56 ± 3.56	17.63 ± 6.32
Single	27	19.07 ± 8.37	27.64 ± 10.27	12.82 ± 5.84	17.82 ± 7.65
Test stat.		− 0.635*	− 1.001*	− 1.319*	− 1.000*
*p*		0.528	0.321	0.194	0.321
*Having child*					
Yes	34	19.15 ± 8.02	26.94 ± 10.53	11.56 ± 3.87	17.12 ± 6.01
No	26	19.81 ± 6.75	28.88 ± 8.87	12.92 ± 5.71	18.50 ± 8.00
Test stat.		− 0.338*	− 0.758*	− 1.103*	− 0.765*
*p*		0.736	0.452	0.275	0.448
*Working status*					
Working	24	20.25 ± 9.64	27.88 ± 12.35	11.04 ± 3.56	15.29 ± 4.01
Not working	36	18.89 ± 5.62	27.72 ± 7.88	12.89 ± 5.33	19.33 ± 7.96
Test stat.		0.625*	0.054*	− 1.609*	− 2.594*
*p*		0.537	0.957	0.113	**0.012**
*Income status*					
Low	15	17.27 ± 7.82	28.40 ± 10.14	12.00 ± 4.99	17.07 ± 7.81
Middle	43	20.33 ± 7.38	27.67 ± 10.00	12.16 ± 4.77	17.95 ± 6.79
High	2	16.50 ± 2.12	25.50 ± 4.95	13.00 ± 5.66	17.50 ± 4.95
Test stat.		1.104**	0.084**	0.038**	0.090**
*p*		0.339	0.920	0.963	0.914
*Place of residence*					
Province	47	20.36 ± 7.89	28.74 ± 10.72^a^	12.15 ± 5.11	17.23 ± 6.89
District	10	17.20 ± 3.91	23.00 ± 3.59^b^	12.20 ± 3.26	20.50 ± 6.57
Town	3	12.33 ± 3.51	28.67 ± 1.53^a^	12.00 ± 4.36	16.00 ± 8.66
Test stat.		5.636**	8.228**	0.002**	1.018**
*p*		**0.038**	**0.004**	0.998	0.368
*Previous surgery status*					
Yes	24	19.08 ± 5.30	28.50 ± 7.85	13.46 ± 5.00	19.63 ± 7.52
No	36	19.67 ± 8.65	27.31 ± 11.01	11.28 ± 4.44	16.44 ± 6.27
Test stat.		− 0.324*	0.459*	1.770*	1.777*
*p*		0.747	0.648	0.082	0.081
*Profession*					
Government employee	20	17.80 ± 8.18	24.60 ± 10.80	11.05 ± 3.58^b^	18.29 ± 7.53
Private sector	14	24.14 ± 8.05	35.07 ± 7.47	12.93 ± 5.58^a^	19.40 ± 7.21
Housewife	15	17.67 ± 5.09	25.40 ± 8.98	11.73 ± 3.97^b^	19.27 ± 9.08
Student	11	18.82 ± 6.15	27.55 ± 7.61	13.73 ± 6.36^ab^	17.72 ± 6.91
Test stat.		2.288**	0.919**	4.145**	1.951**
*p*		0.100	0.438	**0.010**	0.147
*Presence of chronic disease*					
Yes	10	15.20 ± 6.53	20.20 ± 6.71	10.50 ± 2.59	17.60 ± 6.87
No	50	20.28 ± 7.38	29.30 ± 9.67	12.48 ± 5.04	17.74 ± 6.99
Test stat.		− 2.021*	− 2.832*	− 1.823*	− 0.058*
*p*		**0.048**	**0.006**	0.080	0.954

Bold values indicate significant

*DBLS* desire for being liked scale, *BFNE* the brief fear of negative evaluation scale

*Independent-samples *t*-test, **One-way ANOVA

^a,b^No difference between groups with the same letter for each measurement, mean ± sd

Table 4 shows the preoperative and postoperative age variables of the patients and the correlation between the scales. There was a statistically significant and positive correlation between pretest BFNE and DBLS scores at a high level (*r* *=* 0.857; *p* < 0.05) and between posttest BFNE and DBLS scores at a high level (*r* = 0.867; *p* < 0.05). Moreover, there was a weak (*r* = − 0.259; *p* < 0.05) statistically significant and negative correlation between post-test DBLS and age score.

**Table 4:** Correlation between preoperative and postoperative scales of rhinoplasty patients

Variables		Pre-test	Post-test	Age
		DBLS	BFNE	
Pre-test	BFNE			
	*r*	0.857^**^	0.108	− 0.239
	*p*	**<** **0.001**	0.411	0.066
Post-test	DBLS			
	*r*	0.158	0.867^**^	− 0.259^*^
	*p*	0.228	**<** **0.001**	**0.046**

Bold values indicate significant

Pearson correlation coefficient was used

*DBLS* desire for being liked scale, *BFNE* the brief fear of negative evaluation scale

*p < 0.05, **p < 0.01

## Discussion

In the present study, it was determined that the desire for being liked and fear of negative evaluation decreased after surgery in rhinoplasty patients and there was a significant and positive relationship between the desire for being liked and fear of negative evaluation. As a result of the literature review, it is seen that the current study is the first study conducted in these patients. In this respect, the study adds originality to the literature. Due to the limited number of studies, the findings were discussed in line with the literature with similar studies as much as possible.

In the present study, the preoperative desire for being liked and fear of negative evaluation levels of patients living in the provincial center and without chronic diseases were determined to be higher and this difference was significant. It was observed that the desire for being liked after surgery was higher in patients working in the private sector. Furthermore, the levels of fear of negative evaluation after surgery were higher in working people. When the literature was examined, no similar study was found. In Turkey, people have an accepting attitude due to the low population in settlements such as villages and towns and everyone knows each other. In line with these results, it is thought desire for being liked increases in people according to the proportion to the size of the place of residence. Furthermore, those working in the private sector may have to pay more attention to their appearance and may also experience anxiety related to dismissal. These situations may trigger the desire for being liked.

In the present study, it was determined that the desire for being liked decreased as the age of the postoperative patients increased. In a previous study on social appearance anxiety, it was stated that age had a significant effect on the anxiety levels of the participants before and after surgery [13]. In the literature, unlike our study, in a study conducted with female undergraduate students, it was indicated that body appreciation increased with increasing age [19]. Working in a young age group is a limitation that distinguishes this study from the current study. In young people, the desire for being liked can be more intense with the contribution of psychosocial development, and as age progresses, these concerns are replaced by productivity, and the intensity of these desires may decrease [20].

Needs such as receiving positive feedback, and being loved and appreciated play a role in the desire for being liked [8]. With rhinoplasty, a person’s self-confidence and self-esteem can increase, and the long-term mental, emotional, and psychosocial functioning and well-being of that patient can increase [21, 22]. In the present study, it was determined that the desire for being liked, which was present in patients before surgery, decreased significantly after surgery. When similar studies are examined, it is seen that body image and self-esteem increase after surgery [23–25]. Physical appearance is a strong determinant of an individual’s body image, especially for women [26, 27]. These results may suggest that the physical appearance of the patients is as they want after surgery, and in relation to this, patients may think that they are more admired by the individual and society, and as a result, patients’ desire to be admired may decrease.

Self-perception is important in terms of fear of negative evaluation. In this study, it was determined that the fear of negative evaluation that existed in patients before surgery decreased significantly after surgery. In a previous qualitative study, it was determined that individuals were exposed to pressure and negative discourses about the nose and needed comprehensive counseling about the process [28]. In another study, a significant negative correlation was stated between patients’ preoperative social appearance anxiety and postoperative patient satisfaction [12]. In another study, it was reported that patients had better mental status after rhinoplasty surgery [29]. These results suggest that the preoperative fears of the patients due to their physical appearance decreased with the surgery and positively affected their mental state.

In this study, it was observed that as the desire for being liked increased before surgery, the fear of negative evaluation increased and as the desire for being liked decreased after surgery, the fear of negative evaluation decreased. These results show that the patients think that they are liked by themselves and society when their nasal shapes are as they want and that their fear of negative evaluation decreases with the power and self-confidence given by their physical appearance.

## Conclusions and Recommendations

As a result, it was determined that preoperative desire for being liked and fear of negative evaluation of rhinoplasty patients decreased significantly after surgery. Moreover, a high correlation was observed between preoperative and postoperative desire for being liked and fear of negative evaluation. Due to the increase in rhinoplasty today, it is recommended that individuals who will undergo surgery should be psychologically evaluated and, if necessary, psychiatry consultations should be requested and necessary counseling should be provided to individuals. Experimental and quasi-experimental studies with other variables that will reveal the psychological status of rhinoplasty patients are recommended.

## Data Availability

The datasets generated and/or analyzed during the current study are not publicly available but are available from the corresponding author on reasonable request.
